# Idiopathic Pulmonary Fibrosis and Telomeres

**DOI:** 10.3390/jcm11236893

**Published:** 2022-11-22

**Authors:** Alba Mulet, Jaime Signes-Costa

**Affiliations:** 1 Department of Pulmonology, Hospital Clínico, 46010 Valencia, Spain; 2 Respiratory Diseases Research Group, INCLIVA, 46010 Valencia, Spain

**Keywords:** telomere, idiopathic pulmonary fibrosis, genetics

## Abstract

Idiopathic pulmonary fibrosis is an interstitial lung disease of unknown etiology with a highly compromised prognosis and a significant mortality rate within a few years of diagnosis. Despite being idiopathic, it has been shown that telomeric shortening could play an important role in its etiopathogenesis. Mutations in telomere-related genes have been identified, but they are not always present despite telomere shortening. On the other hand, this telomeric shortening has been linked to a worse prognosis of the disease independently of other clinical factors, implying it may serve as a biomarker.

## 1. Introduction

Idiopathic pulmonary fibrosis (IPF) is the most common form of chronic idiopathic diffuse interstitial lung disease in adults. It is a chronic and progressive disease with a high mortality rate that affects mainly elderly individuals, with an average age of diagnosis of 66 years [1]. IPF is defined as occurring spontaneously (idiopathic), and it is the most common type of idiopathic interstitial pneumonia [2], which should be distinguished from interstitial lungs diseases of a specific cause, for example in the context of an autoimmune disease such as connective tissue disease.

IPF is characterized by scarring of the lung that is believed to result from an atypical response to injury of the epithelium. The epithelial damage is thought to be caused by an aberrant fibrotic response with the deposition of dense material, which reduces lung flexibility and its capacity for gas diffusion [3].

The etiopathogenic mechanisms of this disease are not fully understood, with smoking being one of the main risk factors [4,5]. Age is another relevant risk factor, being considered by many epidemiological studies as an aging related disease [6,7]. Both prevalence and incidence increase with age, and it is usually diagnosed after the sixth or seventh decade of life and is more common in men than in women [8].

IPF is recognized as a rare disease with an incidence of around 10 cases per 100,000 per year in Europe and North America [9]. However, its incidence and prevalence have increased in recent years, mainly due to the improvement of the diagnostic methods and the aging of the population [10,11,12].

There are two drugs available: Nintedanib and Pirfenidone, and although both have been shown to slow the progression of the disease, neither is able to stop it completely.

Telomeric shortening has been linked to IPF in multiple studies [13,14]. Telomeres are specialized structures found at the edges of chromosomes that shorten progressively during the life of an individual, which is being proposed as the primary molecular cause of aging. They are known as the mitotic clock, as they mark the entry into cellular senescence [15,16]. Therefore, IPF patients with telomeric shortening have been shown to have a worse prognosis.

In recent years, multiple telomere-related genes have been identified that cause telomere shortening and that are associated in significant percentages with IPF. More specifically, mutations in telomere genes have been found in up to 25% of familial cases and 1–3% of sporadic IPF cases. In addition, in sporadic cases not associated with telomere-related gene mutations, shorter telomeres have been observed compared to people of the same age without the disease, with 10% of patients having telomeres as short as those of telomerase mutation carriers. Telomeric shortening has also been observed in some studies in other lung diseases with fibrosing phenotypes, always giving them a worse prognosis.

The importance of determining the prognosis of the disease has increased with the study of new drugs for this entity and the expanding eligibility for lung transplantation.

The purpose of this review is to summarize the data concerning telomeropathies in relation to IPF.

## 2. Etiopathogenesis

The etiology and pathogenesis of the disease are still unclear, although lung fibroblast and epithelial cell activation, as well as the secretion of fibrotic and inflammatory mediators, have been linked to its development and progression [17]. Moreover, although IPF is, by definition, idiopathic, certain risk factors have been identified [18].

### 2.1. Pulmonary Toxics

Smoking has always been considered the main risk factor, with a percentage of former or current smokers ranging from 41 to 83% [5] (p. 1).

Exposure to stone, metal, wood, and organic dust has also been suggested as a risk factor [19,20].

### 2.2. Gastroesophageal Reflux

It is believed that gastroesophageal reflux may contribute in the form of microaspirations. However, it is difficult to make associations considering its high prevalence in the general population.

### 2.3. Genetic Factors

From 2 to 20% of patients with IPF have another affected family member [21,22,23,24], and transmission is usually autosomal dominant. In addition, several mutations associated with pulmonary fibrosis have been identified. However, most patients with known mutations have additional risk factors, such as exposure to pulmonary toxics. This in turn explains why there are relatives with the same mutation with and without disease [25].

Therefore, although genetic factors represent an important etiopathogenic mechanism, they do not seem to be sufficient or depend, in most cases, on another triggering factor.

Nonetheless, it is considered that the greatest risk factor for IPF is having an affected family member.

### 2.4. Short Telomeres

The capacity to regenerate and repair damaged tissue is progressively reduced with age. One of the mechanisms related to the aging process is telomeric shortening. Therefore, in IPF, as in other diseases with premature telomeric shortening, this phenomenon leads to poor tissue repair, in this case of the pulmonary epithelium, and this drives to lung scarring and the development of pulmonary fibrosis.

Analysis of familial IPF patients with mutations in telomere-related genes showed diminished telomerase activity and prematurely shortened telomere length. Similar results were found in sporadic patients not carrying any mutations when compared to healthy controls [26,27].

These findings suggest that telomeric shortening plays a key role in the etiopathogenesis of IPF, which is not fully explained by genetic mutations, at least not in every case.

On the other hand, it should also be taken into account that telomeric shortening can be caused by exogenous factors, such as oxidative stress or smoking, but can also be due to increased proliferation of immune cells [28,29]. Therefore, it will not always have a hereditary character, as it does in familial cases with identified mutations. Sometimes it may have an acquired role that also contributes to the development of the disease. Also, it should be noted that telomeric shortening in the parents is transmitted to the offspring, even if they do not have a mutation in related genes.

## 3. What Are Telomeres?

Telomeres are non-coding repetitive sequences of nucleotides (hundreds to thousands of TTAGGG tandem repeats) found at the ends of chromosomes that protect them from erosion. At each cell division, these repeats are lost, thus shortening the telomeres progressively [30].

Telomere shortening is considered the primary molecular cause of aging since shortened telomeres limit the replicative capacity of stem cells, blocking the regenerative capacity of tissues, and thus causing cellular senescence. This leads to the development of age-related diseases [31].

**Figure 1:**
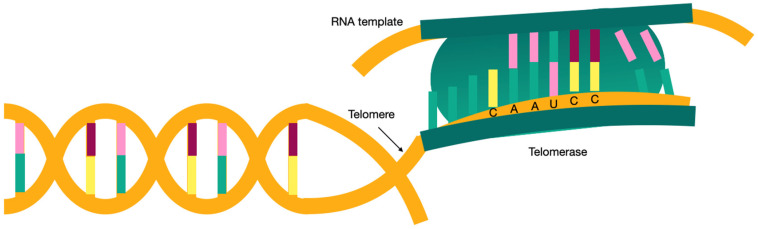
Telomerase.

Telomerase is a reverse transcriptase that is able to elongate telomeres de novo by adding TTAGGG repeats to chromosome ends [32] after mitosis, thus preventing the loss of encoded information (Figure 1). Telomerase is active in embryonic stem cells; however, after birth it is silenced in most cells, causing the telomeres to shorten over the years. This shortening of the ends of the chromosomes triggers the activation of a p53-dependent DNA damage response that triggers senescence or apoptosis of the cells [33,34].

Several recent studies have demonstrated the relationship between telomeric shortening and loss of cellular immune function, known as immunosenescence.

When there are mutations in genes related to telomere maintenance, the corresponding diseases are known as telomeropathies. Classic telomeropathies include: Hoyeraal-Hreidarsson syndrome, dyskeratosis congenita, pulmonary fibrosis, aplastic anemia, and liver fibrosis.

A variety of experimental and genetic studies support the hypothesis that telomere shortening contributes to the pathogenesis of IPF; however, it does not seem to be enough. In a mouse model with telomerase deletion, no phenotypic changes were seen. However, after several generations, mice with short telomeres developed degenerative diseases with phenotypic changes related to aging [35,36].

Therefore, telomeric shortening plays a key role in the etiopathogenesis of IPF, but a second hit, such as smoking, viral infections, or gastroesophageal reflux, is necessary to contribute to the onset of IPF.

In addition, it is known that telomeric length, which is known to be an inheritable trait from the progenitors [37], will therefore contribute to the heritability of the disease.

## 4. Genetics in IPF

In recent years, there has been growing evidence that genetic factors play an important role in IPF. In addition, the fact that there are several relatives affected by IPF in the same family suggests a genetic mechanism. However, mutations in these genes are not exclusive to familial forms they have also been found in sporadic cases [38,39].

The genes most frequently affected by the mutations are as follows (they are all telomere integrity genes) (Table 1):

On the other hand, there are the surfactant-encoding genes, which are much rarer:

- SFTPC
- SFTPA2

There are more familial or sporadic patients with telomeric shortening than with detected mutations; therefore, it is believed that most of the genetic mechanisms remain unknown [14] (p. 1) and that telomere shortening is a more generalized feature of IPF.

### Familial Pulmonary Fibrosis

Familial pulmonary fibrosis (FPF) is considered when there are two or more affected members, and it represents up to 20% of the cases of IPF, depending on the series [40,41,42].

The demographic and clinical characteristics of patients with FPF are similar to those of patients with sporadic disease, except for their younger age at diagnosis [43], being diagnosed about 3.5 to 12 years earlier [44]. In addition, they tend to have a worse prognosis because progression occurs earlier.

It exhibits a wide range of pulmonary fibrosis phenotypes, being in most cases idiopathic pulmonary fibrosis. However, there can also be different diagnoses in the same family: hypersensitivity pneumonitis, desquamative interstitial pneumonia, and pleuropulmonary fibroelastosis, among others.

## 5. When to Ask for Telomere Length?

Telomere length can be determined by different methods. These include Southern blot, quantitative PCR (Polymerase Chain Reaction), and Flow-FISH (flow cytometry with fluorescence in-situ hybridization), which are the most frequently used since they don’t require a large amount of DNA. It can also be measured with TRF (Terminal Restriction Fragment) analysis, STELA (single telomere length analysis), which was designed to measure telomeres on individual chromosomes, and TeSLA (telomere shortest length assay), the newest technique, but it is usually used only for research [45].

In addition, telomere length can be determined in several places: in peripheral blood leukocytes, the most classically studied method, or, as recently described, with an oral swab harvesting epithelial cells from the cheek. There is no standard technique to measure telomere length, but these two techniques are the most accessible. Moreover, telomere length can also be measured in lung epithelial cells, specifically, alveolar type 2 cells. In these cells, it has been determined in different areas of the lung, with no differences found between areas regardless of the typical fibrosis pattern (apicobasal gradient) [46,47]. Several studies have shown that all methods are equivalent [48]. Therefore, the first two techniques can be used on a day-to-day basis, as they are less invasive, and the last one can be reserved for research-related issues.

**Figure 2:**
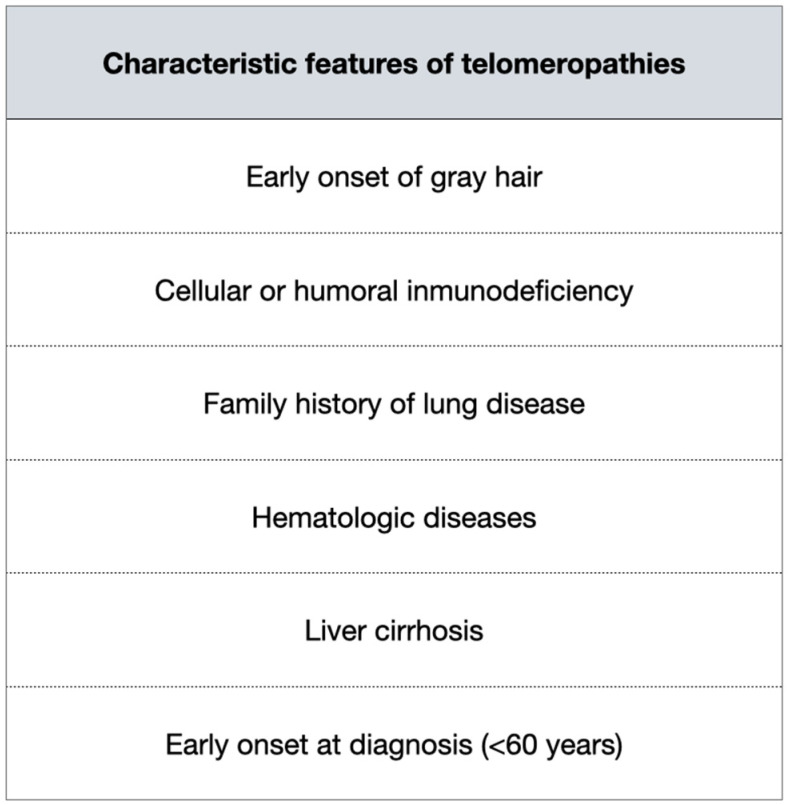
Characteristic features of telomeropathies.

On the other hand, all these tests and, above all, the genetic testing are expensive and/or not always available, so they are not recommended at this time if no genetic disease is suspected. There are certain characteristics of the patients that should make us think of telomeropathies, and therefore we should request further testing (Figure 2).

### Screening for Relatives

The importance of screening relatives lies in the fact that interstitial alterations have been observed in up to 25% of first-degree relatives of patients with IPF [49,50,51,52,53]. There is also an increased risk of death by pulmonary fibrosis in first- and second-degree relatives.

Inherited short telomere lengths in the absence of telomerase mutations have been reported previously [54]. This could explain the high probability of pulmonary fibrosis in relatives, even if there is no mutation. Moreover, as previously mentioned, telomeric shortening is a heritable trait without requiring a mutation in a telomere-related gene, and it has been shown that over the generations it progressively shortens even more.

Currently, there are no guidelines for what to do when a genetic form of IPF is suspected. It should also be noted that the disease does not usually manifest itself, even in inheritable cases, before the age of 40 [25] (p. 2). Therefore, screening younger people would be questionable.

Accordingly, as mentioned above, due to the limited availability of the tests, it must be carefully considered in which contexts to carry out telomeric/genetic studies. A clear context for doing counselling would be when a mutation in telomere or surfactant-related genes has been detected in the index case. In this case, genetic counseling should be given to first-degree relatives who want it.

On the other hand, with the increasing importance of genetics in medicine, it might be reasonable that all individuals with IPF are offered genetic counseling and it seems also essential to counsel future generations of parents with telomeric shortening or related mutations, especially to avoid substances that are harmful to the lung and that have been shown to be particularly sensitive to telomeric dysfunction, like smoking or other toxics [55].

## 6. Telomeres Relevance in Treatment

Currently, there are only two drugs approved for IPF: Pirfenidone, which is an antifibrotic agent that inhibits transforming growth factor beta (TGF-b)-stimulated collagen synthesis and decreases extracellular matrix and fibroblast proliferation, and Nintedanib, which is a receptor blocker of multiple tyrosine kinases that collaborate in the synthesis of profibrogenic growth factors. Both drugs are only capable of slowing down the disease without stopping it completely. On the other hand, the incidence and prevalence of IPF have been increasing, as the population ages, and there is an urgent need for improved therapies.

There is no specific treatment for IPF patients with telomeropathy. However, in the future, it would be an interesting field to study in order to choose a more personalized treatment for the patient.

Newton et al. [56] measured telomere length in PANTHER-IPF patients and found that telomere length was a good marker to determine those patients who would have a poor response to immunosuppressive therapy. This has also been seen in patients with short telomeres who are transplanted and given long-term immunosuppressive treatment who have worse survival than patients with preserved telomeres [57]. That is, adverse reactions occurring in IPF patients treated with immunosuppressants are related to telomeric length. Therefore, telomere measurement could be of help in certain patients when deciding on a more personalized treatment.

It has also been studied whether to use the maintenance of telomere length as a target for therapy. Townsley et al. studied the use of synthetic danazol, the sex hormone, to attenuate the accelerated telomere attrition since, in tissue culture and animal models, sex hormones regulate expression of the telomerase gene. The results showed that almost all the patients (11 of 12; 92%) had a gain in telomere length at 24 months as compared with baseline [58]. A recent study has also investigated a new mechanism for increasing telomere length by transferring telomeres to T cells from other types of immune cells, independent of telomerase activity. The researchers have found that some cells can acquire telomeric DNA from other cells transported through extracellular vesicles. For the time being, it has begun to be explored as an immune system enhancer [59].

Therefore, this is a field with possibilities for new, more personalized therapeutic approaches in the era of precision medicine.

## 7. Prognostic Biomarkers

The mean age-adjusted telomere length in IPF patients was significantly shorter than that in age-matched controls. In addition, telomeric length has been found to be an independent risk factor in several studies [60,61], being associated with worse survival. This remains true despite eliminating patients carrying mutations in telomerase-related genes.

It has also been shown to be associated with a worse post-transplant prognosis and a greater number of associated complications, both due to the use of immunosuppressive medication, which, as previously mentioned, is related to adverse effects in these patients, as well as increased mortality related to shorter telomeres. It is suggested that poorer transplant survival may be related to higher rates of chronic lung allograft dysfunction and a shorter time to its onset [57] (p. 6).

On the other hand, although age in itself is a poor prognostic factor for IPF in general (the older the patient, the worse the prognosis), the opposite has been seen in patients with telomeric shortening. The younger they are at diagnosis, the worse their evolution will be. This can be explained by the fact that the earlier the fibrotic disease presents, the faster the lung aging process develops [48] (p. 4).

Telomere length is a biomarker capable of preventing a worse prognosis and therefore, can help to stratify risk and decide on a referral for transplantation. Therefore, its measurement is recommended as part of the pretransplant workup of patients with IPF.

On the other hand, telomeric shortening has also been shown to be associated with increased risk of infection and increased mortality when infections occur [62,63]. Hence, patients are going to be more susceptible to infections, and furthermore, they are going to have a worse prognosis when they do get them.

In IPF, follow-up is performed with pulmonary function tests, especially forced vital capacity (FVC). FVC decline is predictive of mortality in patients with IPF. However, there is currently no useful biomarker in clinical practice to predict this decline. Consequently, as telomeric shortening predicts a susceptibility to develop progressive fibrosing disease, it could be used together with other biomarkers as a prognostic factor for survival, to help us know the patient’s evolution and decide on possible therapeutic options.

## 8. Telomeres in Others Interstitial Diseases

Several studies have shown that telomeric shortening does not occur only in IPF but has also been related to other types of interstitial lung diseases, such as unclassifiable diseases, hypersensitivity pneumonitis, and pleuroparenchymal fibroelastosis, among others [64].

In these diseases, telomeres are shorter compared to controls; however, there are differences between each of the entities. Above all, a greater shortening has been seen in the case of IPF, implying a genetic factor, innate rather than acquired as in the other entities, likely acquired through increased oxidative stress or inflammatory responses [27] (p. 2).

Moreover, telomere-related mutations have been identified in patients with non-IPF fibrosis. However, the one consistent phenotype seen across all genetic mutations is progressive deterioration. So, although clinical diagnosis is important for therapeutic and prognostic decisions, the identification of telomeric shortening or mutation in its related genes predicts a similar pathogenic mechanism, which manifests in a progressive fibrosing phenotype and confers a worse prognosis.

## 9. Conclusions

IPF is an aging-related interstitial lung disease of unknown etiology with a progressive evolution and an often fatal outcome. Telomere pathology has been linked to epithelial cell senescence, which has been identified in the lining fibroblast foci of IPF lung.

Pulmonary fibrosis is the most frequent manifestation of telomeropathies. Several studies have shown that telomeric shortening is a risk factor for the disease, regardless of whether or not mutations in specific genes are identified.

Age- and sex-adjusted telomere length is shorter in IPF patients than in the general population, in both the familial and sporadic forms of pulmonary fibrosis, and regardless of having mutations in related genes. Moreover, telomeric shortening is more marked in patients who have a worse prognosis.

The clinical course of IPF is variable, and no reliable prognostic biomarkers are currently available. Therefore, the study of telomeric shortening in IPF provides prognostic data, functions as a biomarker, helps us prevent complications related to certain medications, and could help us in the risk stratification prior to transplantation.

It is important to know the characteristic features of telomeropathies in order to identify those patients susceptible to telomere measurement and/or genetic study, as for the time being, these tests are not yet widely available and are expensive.

Modulation of telomerase expression and telomere length may be a therapeutic tool for the treatment of these disorders. Future research should focus on the molecular mechanism underlying the shortening of telomere length in IPF.

## Figures and Tables

**Table 1:** Telomere-related genes in IPF.

**Telomerase reverse transcriptase (TERT)**	The coding gene for the telomerase reverse transcriptase. It is responsible for 15% of familial pulmonary fibrosis (FPF). There are gender differences in this mutation, with females being 11.9 years older at diagnosis than males.
**Telomerase RNA Component (TERC)**	The gene encoding the telomerase RNA component.
**Regulator Of Telomere Elongation Helicase 1 (RTEL1)**	Regulator of Telomere Elongation Helicase 1. The established locus for dyskeratosis congenita.
**Poly(A)-Specific Ribonuclease (PARN)**	Poly(A)-specific Ribonuclease Deadenylation Nuclease.
